# Relationship between FAT/CD36 protein in skeletal muscle and whole-body fat oxidation in endurance-trained mice

**DOI:** 10.20463/jenb.2016.0057

**Published:** 2016-12-31

**Authors:** Jisu Kim, Kiwon Lim

**Affiliations:** 1 Department of health and exercise science, Korea National Sport University, Seoul Republic of Korea; 2 Physical Activity and Performance Institute (PAPI), Konkuk University, Seoul Republic of Korea

**Keywords:** FAT/CD36, CPTІ, Long-chain fatty acid proteins, Endurance exercise

## Abstract

**[Purpose]:**

We investigated the effects of endurance training on the expression of long-chain fatty acid transport proteins in the skeletal muscle and whole-body fat oxidation during endurance exercise.

**[Methods]:**

Seven-week-old male ICR mice (n = 12) were divided into 2 groups, namely, Sed (sedentary; non-trained) and Tr (endurance-trained) groups. The Tr group was adapted to treadmill training at a fixed intensity (15 m/min, 8° slope) for 3 days. Next, the exercise intensity was increased while maintaining the 8° slope. In the last week of training, the exercise intensity was set at 25 m/min for 50 min (about 70–75% maximal oxygen uptake for 4 weeks). After the protocol ended, the mice were sacrificed, and tissues were collected for western blot analysis.

**[Results]:**

Four weeks of endurance training resulted in a significant increase in the protein levels of FAT/CD36 and CPTІ. The FAT/ CD36 protein level in the Tr group was about 1.3-fold greater than that in the Sed group (p < 0.01). Furthermore, the increased CPTІ indicated higher activity (19% upregulation) in the Tr group compared to the Sed group (p < 0.05). The FAT/CD36 protein level and the estimated whole-body fat oxidation rate during 1-h exercise were found to be significantly correlated (r = 0.765, p < 0.01).

**[Conclusion]:**

We suggest that the increase in FAT/CD36 protein in skeletal muscle by endurance training might be positively associated with whole-body fat oxidation, which might enhance endurance exercise capacity.

## INTRODUCTION

Muscle adaptations to aerobic endurance training include increased capillary density and mitochondrial number and size^1^. Aerobic endurance training increases the activity of the enzymes of the tricarboxylic acid cycle and of other oxidative enzymes (hormone-sensitive lipase, catecholamines, β-oxidation enzymes, etc.), with a concomitant increase in the capacity to oxidize both fat and carbohydrate. The concentrations of long-chain fatty acid (LCFA) transport proteins increase as well, which might facilitate the uptake of free fatty acids (FFAs) across the sarcolemma^2^. A consequence of and a likely contributing factor to the improvement of exercise capacity after endurance training is the metabolic shift to a greater use of fat and a concomitant sparing of glycogen^3^. Therefore, fat oxidation is very important for endurance athletes. Some evidence suggests that increases in plasma FFA concentration can suppress the rate of muscle glycogen utilization. This action might theoretically be beneficial, because muscle glycogen depletion is one of the prime causes of fatigue^4^.

LCFA transport proteins (FAT/CD36, CPTІ, CPTІІ, FATP, FABPc, and FABPpm) have been reported to comprise a major component of fat metabolism, because they facilitate the transport of FFAs into cells and mitochondria^5, 6^. Among them, FAT/CD36 and CPTІ have key roles in muscle fuel selection, exercise performance, and training-induced muscle fatty acid oxidation adaptation in humans^7, 8^. Recently, McFarlan et al.^9^ reported FAT/CD36 overexpression in mice, which occurred independent of mitochondrial changes after the upregulation of exercise-induced fatty acid oxidation. However, FAT/CD36-knockout (KO) mice showed reductions in fatty acid transport (-21%) and oxidation (-25), intramuscular lipids (less than or equal to -31%), and hepatic glycogen (-20%) compared with wild-type mice. In an earlier study, untrained women were subjected to 6 weeks of high-intensity training (14-min cycling bouts at 90% VO2 peak, separated by 2 min of rest), and muscle biopsies revealed significantly increased maximal oxygen uptake at 2 and 6 weeks. In addition, the expression of FAT/ CD36 increased at the whole-muscle (10%) and mitochondrial levels (51%)^10^. Tunstall et al.^11^ reported that in healthy, untrained human subjects subjected to endurance training in the form of cycle ergometer training at 63% ± 2% (VO2 peak, 104 ± 14 W) for 60 min/day for 9 days, fat oxidation increased by 24%. Moreover, training increased the expression of FAT/CD36 and CPTІ mRNA in skeletal muscle. Similarly, FAT/CD36 protein was also upregulated by exercise training.

Meanwhile, in a different study, we reported that 4 weeks of endurance training (70~75% maximal oxygen uptake) did not change the resting metabolic rate (oxygen uptake and respiratory exchange ratio) for 24 h. We had used the open-circuit calorimetry system, which uses a mass spectrometer and has a considerably higher precision than that of existing gas analyzers. Nevertheless, the weight and abdominal fat were found to be reduced, because the training was shown to promote fat oxidation (33%) during exercise for 1 h^12^. However, the relationship between the expression of LCFA transport proteins in skeletal muscle and the alteration of whole-body fat oxidation during endurance exercise remains unclear. Therefore, the purpose of this study was to ascertain the effects of endurance training on the expression of LCFA transport proteins in skeletal muscle and whole-body fat oxidation during endurance exercise.

## METHODS

### Animals and training protocol

In our previous study^12^, 7-week-old male ICR mice (n = 12) were obtained from Orient Bio Inc. (Seongnam, Korea). The mice were divided into 2 groups: Sed (sedentary; non-trained) and Tr (endurance-trained) groups. Training was commenced at a specific time-period, with a frequency of 5 times a week for 4 weeks. The Tr group was adapted to treadmill training (treadmill from Daejong, Systems, Korea) at a fixed intensity (15 m/min, 8° slope) for 3 days. The exercise intensity was then increased while maintaining the 8° slope. In the last week of training, the exercise intensity was set at 25 m/min for 50 min. Mice were sacrificed on the day following the end of the protocol, and tissues were collected for western blot analysis. The results of whole-body fat oxidation and skeletal muscle findings were sampled from our earlier study^12^. However, the purpose of this study was to achieve findings different from those of that study using the data obtained earlier. The detailed exercise protocol and sampling and analysis methods of this study have been published^12^.

### Protein extraction and western blot analysis

Soleus muscle tissue was homogenized using a mortar and TissueRuptor (Germany, Qiagen). The soleus tissue (20 mg) was homogenized in 700 μL RIPA lysis buffer (EzRIPA Lysis, ATTO Biotechnology, Sungnam, Korea). Lysates were centrifuged at 12,000 rpm at 4°C for 15 min. The protein concentration of the lysates was determined with the GenDEPOT Protein Assay plus Reagent (Gen-Depot Laboratories, USA) using bovine serum albumin as the standard.

Total protein (25 μg per lane) was separated by 12% sodium dodecyl sulfate-polyacrylamide gel electrophoresis at 100 V for 120 min and transferred to polyvinylidene difluoride membranes (Millipore, Billerica, MA, USA) at 100 V for 150 min. The membranes were blocked for 1 h at room temperature with phosphate-buffered saline (PBS; HyClone Laboratories, USA) containing 5% skim milk (Difco, USA) and then washed 3 times (for 5, 5, and 15 min each) with PBS with 0.1% Tween 20 (PBS-T). After overnight incubation at 4°C with primary antibodies against FAT/CD36 and CPTІ (Santa Cruz Biotechnology, USA), the membranes were washed with PBS-T and incubated with a horseradish peroxidase-conjugated secondary antibody for 1 h at room temperature. Immunodetection was carried out with ECL detection reagent (Amersham Biosciences, Uppsala, Sweden). All figures showing the results of quantitative analysis (Image J, National Institutes of Health) include data from at least 3 independent experiments.

### Statistical analysis

Data are given as mean ± standard deviation. All statistical analyses were performed with SPSS version 19.0 software (SPSS, Inc., Chicago, IL, USA). The expression levels of FAT/CD36 and CPTІ were analyzed by unpaired t-tests between groups. Linear relationships between key variables were tested using Pearson’s correlation coefficients. Differences were considered significant at p < 0.05.

## RESULTS

### FAT/CD36 and CPTІ expression

**Figure 1.**
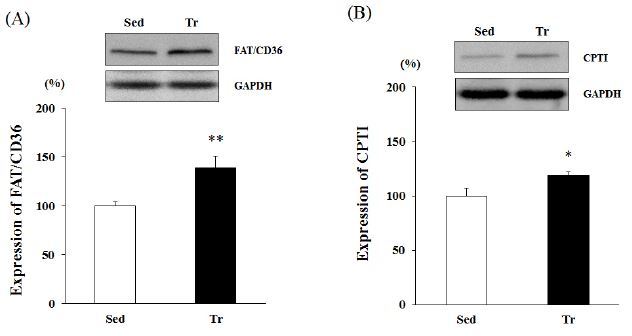
**Western blot analysis of the expression levels of FAT/CD36 and CPTІ in skeletal muscle (soleus)**. Results are expressed as relative activity (%) in the Tr (endurance training) group compared with the Sed (sedentary) group. A, FAT/CD36 expression level. B, CPTІ expression level. Each bar represents the mean ± standard deviation. *, ** vs. Sed group (*p < 0.05, **p < 0.01).

Western blot analysis was performed using the proteins obtained from skeletal muscle (soleus). Four weeks of endurance training resulted in a significant increase in the protein expression of FAT/CD36 and CPTІ. The FAT/ CD36 protein level in the Tr group was about 1.3-fold greater than that in the Sed group (p < 0.01; Figure 1 A). Moreover, the increased CPTІ levels indicated higher activity (19% upregulation) in the Tr group than in the Sed group (p < 0.05; Figure 1 B).

### Correlations between whole-body fat oxidation and LCFA gene expression

**Figure 2.**
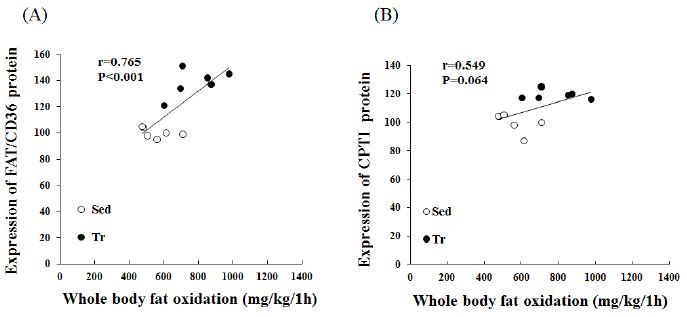
**Pearson’s correlation coefficients between LCFA proteins and whole-body fat oxidation rates for 1 h during exercise (at 70–75% VO2 max)**. A, calculated Pearson’s correlation coefficients between estimated whole-body fat oxidation rates (mg/kg/1 h) and FAT/CD36 expression (%). B, calculated Pearson’s correlation coefficients between estimated whole-body fat oxidation rates (mg/kg/1 h) and CPTI expression (%) in skeletal muscle (n = 12).

We further analyzed the expression of LCFA-related genes to determine their potential relationship with whole-body fat oxidation (mg/kg/1 h). A significant correlation (r = 0.765, p < 0.01) was found between the estimated whole-body fat oxidation rate and the FAT/CD36 protein expression in skeletal muscle (Figure 2A). In addition, CPTІ correlated positively with whole-body fat oxidation (mg/kg/1 h); however, this was not statistically significant (r = 0.549, p < 0.064).

## DISCUSSION

In the present study, we investigated the effects of endurance training on the expression of LCFA transport proteins in skeletal muscle and correlations with whole-body fat oxidation. We observed that after the endurance training, total muscle FAT/CD36 and CPTІ contents increased, and FAT/CD36 and CPTІ correlated positively with whole-body fat oxidation. These results suggest that an increase in skeletal muscle fatty acid oxidation after endurance training was partly related to changes in LCFA protein content. In addition, using the open-circuit method, we observed greater fat oxidation in the Tr group compared to the Sed group according to the assessment of energy metabolism during exercise^12^. This result might be attributed to the increased action of the enzymes of the fat metabolism pathway as a result of 4 weeks of training.

Interestingly, the increased fat oxidation rate during 1 h of exercise correlated with both the whole-body fat oxidation rates and FAT/CD36 with CPTІ protein content, suggesting an important role of FAT/CD36 and CPTІ in LCFA transport during exercise. The LCFA transport proteins FAT and CD36 have been isolated from adipocytes^13^ and plasma membranes^8, 14, 15^. They have been shown to influence the transport of LCFAs across the plasma membrane in both animal and human skeletal muscle. Recently, FAT/CD36 has also been reported to be present on mitochondrial membranes of rat^16^, mouse^9^, and human^17^ skeletal muscle. FAT/CD36 has been shown to be required for palmitate and palmitoyl-carnitine oxidation in skeletal muscles using human and animal models. Thus, FAT/CD36 has been proposed to facilitate the transport of LCFA-carnitine to CPTІ.

The importance of FAT/CD36 in resting and dieting situations remains unclear. However, the present findings demonstrated that training induced an increase in FAT/ CD36 protein expression, which is correlated with the rate of palmitate oxidation in rat skeletal muscle^16^. The metabolic effect of FAT/CD36 ablation under basal conditions was consistent with previous reports demonstrating ablation of CD36-activated circulating fatty acids, impaired fatty acid transport into muscle, and decreased muscle fatty acid oxidation^18-20^. These alterations were associated with better glucose tolerance, increased muscle insulin sensitivity^21^, and increased rates of glucose oxidation, as well as decreased hepatic but not muscle glycogen content. Thus, under basal conditions, FAT/CD36 ablation shifts muscle fuel selection to an increased reliance on glucose utilization. McFarlan et al.^9^ reported that in FAT/ CD36-KO mice, the reduced fatty acid transport into muscle induced a profound shift in fuel selection. Specifically, glycogen utilization by muscle was markedly enhanced and hepatic glycogen was substantially depleted (25%). This is because the plasma membrane fatty acid transporter CD36 is a key component of the molecular machinery required for regulating skeletal muscle fuel selection during acute metabolic exercise, as well as influencing energy metabolic substrate utilization during exercise.

In conclusion, our data confirmed that FAT/CD36 and CPTІ expression significantly increased in skeletal muscle after 4-week endurance training. We also found a positive relationship between FAT/CD36 protein level in skeletal muscle and whole-body fat oxidation during exercise after a 4-week training period. The increase in FAT/CD36 protein in skeletal muscle by endurance training might be positively associated with whole-body fat oxidation, which might in turn enhance endurance exercise capacity.
